# How to Catch a Falsifier: Comparison of Statistical Detection Methods for Interviewer Falsification

**DOI:** 10.1093/poq/nfab066

**Published:** 2022-02-15

**Authors:** Silvia Schwanhäuser, Joseph W Sakshaug, Yuliya Kosyakova

**Affiliations:** 1 PhD candidate with the Institute for Employment Research (IAB), Nuremberg, Germany; and the University of Mannheim, Baden-Württemberg, Germany; 2 professor of statistics with the Institute for Employment Research (IAB), Nuremberg, Germany; the University of Mannheim, Baden-Württemberg, Germany; and Ludwig-Maximilian University of Munich, Munich, Germany; 3 postdoctoral senior researcher and associate lecturer with the Institute for Employment Research (IAB), Nuremberg, Germany; the University of Mannheim, Baden-Württemberg, Germany; and Otto-Friedrich University of Bamberg, Bamberg, Germany

## Abstract

Deviant interviewer behavior is a potential hazard of interviewer-administered surveys, with interviewers fabricating entire interviews as the most severe form. Various statistical methods (e.g., cluster analysis) have been proposed to detect falsifiers. These methods often rely on falsification indicators aiming to measure differences between real and falsified data. However, due to a lack of real-world data, empirical evaluations and comparisons of different statistical methods and falsification indicators are scarce. Using a large-scale nationally representative refugee survey in Germany with known fraudulent interviews, this study tests, evaluates, and compares statistical methods for identifying falsified data. We investigate the use of new and existing falsification indicators as well as multivariate detection methods for combining them. Additionally, we introduce a new and easy-to-use multivariate detection method that overcomes practical limitations of previous methods. We find that the vast majority of used falsification indicators successfully measure differences between falsifiers and nonfalsifiers, with the newly proposed falsification indicators outperforming some existing indicators. Furthermore, different multivariate detection methods perform similarly well in detecting the falsifiers.

Interviewer-administered surveys are often treated as a superior form of data collection, for example, concerning response rates, communication with respondents, and administration of long questionnaires (Groves et al. 2009; Olson et al. 2020). By encouraging respondents’ participation, answering their queries, and ensuring questionnaire completion, interviewers play a vital role for survey quality. However, previous research has emphasized numerous possible survey errors attributable to the interviewer (Fowler and Mangione 1990; Groves 2004). The falsification of survey interviews is one specific and understudied error associated with the interviewer. Interviewer falsification may take various forms, such as intentional miscoding of respondents’ eligibility status or answers, deviations from instructions, and, the most severe form, the fabrication of complete interviews (AAPOR 2003). Although empirical evidence suggests that complete falsification is a rare event (Blasius and Friedrichs 2012), even small amounts of undetected fraudulent data can severely bias survey estimates, particularly in multivariate analyses (Schräpler and Wagner 2005; Landrock 2017; DeMatteis et al. 2020).

Accordingly, the ongoing improvement of strategies for detecting falsified interviews is crucial for optimizing and ensuring data quality. Statistical detection approaches offer an effective and cost-efficient means of complementing commonly used nonstatistical detection strategies (e.g., monitoring and reinterview procedures), by making those actions more focused on suspicious interviewers. Correspondingly, an increasing number of statistical detection methods (e.g., cluster analysis) and falsification indicators (e.g., interview duration) have been developed to identify potentially fraudulent interviewer behavior (Stokes and Jones 1989; Hood and Bushery 1997; Murphy et al. 2004; Li et al. 2011; Birnbaum 2012; Bredl, Winker, and Kötschau 2012; Blasius and Thiessen 2013; Slomczynski, Powalko, and Krauze 2017; Cohen and Waren 2021).

The multitude of proposed statistical methods, however, makes it difficult to identify the method(s) best suited for detecting falsifications. Empirical evaluations and comparisons of identification methods and falsification indicators using real-world data are rare, as most studies rely on experimental data (Menold et al. 2013; Storfinger and Winker 2013) or small datasets with few falsified interviews (Bredl, Winker, and Kötschau 2012). Moreover, studies have mainly focused on evaluating only one method or a few falsification indicators. Using survey data including around 600 verified falsifications for person-level and household-level interviews, we address the challenges practitioners face when deciding on an appropriate detection strategy by empirically examining and comparing the performance of different statistical detection methods and falsification indicators. First, we test different multivariate detection strategies, including cluster analysis under different clustering algorithms, as well as a newly developed detection method we term the *meta-indicator*. Using different accuracy measures, we assess the performance of these detection tools. Second, we introduce some new falsification indicators, which are shown to be useful for the data used. Third, we compare the explanatory power of single indicators and test their directional assumptions pointing to suspicious interviewer behavior.

## Detecting Falsifiers: Previous Research

### Interviewer Falsification in Practice

There are various forms of interviewer falsification. The most blatant is the fabrication of entire interviews. A related form is the partial falsification of interviews. Further forms of falsification include interviewers deviating from prescribed selection rules, interviewing any available person instead of the—maybe unwilling—target respondent, misclassifying noncooperative target persons as ineligible cases, or deviating from the intended interview mode (AAPOR 2003; DeMatteis et al. 2020). Additionally, the intentional miscoding of a given answer to filter questions (Eckman et al. 2014; Kosyakova, Skopek, and Eckman 2015), in order to shorten the interview, is considered falsification.

The application of detection methods to identify falsifiers is an essential part of the quality control process. Traditionally, survey organizations use a wide range of nonstatistical methods as part of their control routines, for example, validation of survey data with administrative data, interview monitoring, and reinterview routines (Hauck 1969; Koch 1995; Jesske 2013). Newer approaches use GPS data to verify interviewer travel routes, digital capture tools to collect screenshots or photos of the interview location (Finn and Ranchhod 2015; Thissen and Myers 2016; Wagner, Olson, and Edgar 2017), or rapid feedback systems to improve monitoring (Edwards, Sun, and Hubbard 2020). Nevertheless, these procedures have some limitations. For example, validation with administrative data is seldom possible and monitoring face-to-face interviews often requires respondent consent to record the interview. Reinterview methods are costly and can lead to erroneous suspicion against honest interviewers if respondents misremember the encounter (DeMatteis et al. 2020).

### Statistical Methods for Detecting Interviewer Falsification

Statistical detection methods are increasingly being used to detect potential interviewer falsification, capitalizing on the notion that falsifiers tend to produce anomalous patterns in the survey data. Such methods aid in flagging suspicious interviewers, enabling more targeted monitoring and cost-efficient use of reinterviewing. Although the methods often share similar underlying assumptions, they differ in their concrete implementation and can be divided into two—sometimes overlapping—approaches: (1) data-driven approaches, focusing on conspicuous patterns in the data, and (2) behavior-oriented approaches, focusing on specific data patterns corresponding to assumptions regarding falsification behavior.

Data-driven approaches include outlier analysis, statistical modeling, and duplicate analysis. *Outlier analysis* compares outcomes of individual interviewers with the average outcome in the survey data using distance measures, or identifies outlying interviewers based on unusual or rare response patterns and response combinations (Murphy et al. 2004; Porras and English 2004). *Statistical modeling* relies on characteristics of interviewers (e.g., tenure or individual response rates) and parameters from previous interviews or waves (e.g., response likelihood) to model the falsification likelihood for an interview (Biemer and Stokes 1989; Li et al. 2011). More recently, supervised machine-learning algorithms (Birnbaum 2012; Weinauer 2019) and multilevel models (Sharma and Elliott 2020) have been utilized to classify possible falsifiers. *Duplicate analysis* flags identical response patterns occurring in multiple interviews (Slomczynski, Powalko, and Krauze 2017), “near-duplicates,” that is, data with an unusually high correspondence of identical response values (Koczela et al. 2015; Kuriakose and Robbins 2016), or duplicate response patterns across same-scaled item batteries (Blasius and Thiessen 2013), and is additionally suitable for identifying fraud by supervisors or other higher administrative-level staff.

The behavior-oriented approaches—which are of primary interest for our empirical investigation—focus on systematic differences in response behavior between real and falsified interviews. These differences are measured by *falsification indicators* (including, for example, the fraction of acquiescent responding, extreme responding, or item nonresponse), which rely on assumptions regarding the rational behavior of falsifiers. While falsification indicators can be analyzed separately, they are often analyzed jointly using multivariate methods to increase the reliability of the detection results. Bredl, Winker, and Kötschau (2012) used *cluster analysis* to divide suspicious and unsuspicious interviewers into subgroups based on a selection of falsification indicators (also see Winker et al. 2013; de Haas and Winker 2016; Bergmann, Schuller, and Malter 2019). Compared to the aforementioned data-driven methods, falsification indicators and cluster analysis can be applied to every survey regardless of the topic or population. It does not require prior knowledge on variables prone to outliers and unlikely response combinations, or the falsification likelihood and actual falsification status. Nevertheless, given the variety of clustering algorithms to choose from, it is unclear which are most suitable for identifying falsifiers in practice. Interpreting the results is not always straightforward since the optimal number of clusters is usually unknown: a two-cluster solution (suspicious versus nonsuspicious interviewers) is prone to falsely suspecting many interviewers, whereas allowing for more clusters may lead to ambiguous interviewer groups.

### Falsification Indicators

Falsification indicators aim to identify patterns produced by fraudulent interviewer behavior. Hence, they are rooted in the idea of the rational behavior of falsifiers who try to maximize their monetary benefit and minimize their time expenditure and effort, while trying to remain undetected (Menold et al. 2013). The majority of falsification indicators are analogous to data quality indicators used to study suboptimal respondent behaviors (e.g., straightlining, primacy/recency effects), but the difference is that each respondent-level outcome is aggregated to the interviewer level to indicate suspicious behavior attributable to the interviewer. Various indicators have been successfully used in quality control processes (Stokes and Jones 1989; Bushery et al. 1999; Turner et al. 2002) and tested on data with known falsifications (Schräpler and Wagner 2005; Bredl, Winker, and Kötschau 2012; de Haas and Winker 2016). In the following paragraphs, we present the fabrication indicators used in this paper.

For example, time stamps are used to identify interviewers with suspiciously short interviews (Bushery et al. 1999; Li et al. 2011) and a high proportion of missing telephone numbers could indicate a falsifier’s effort to prevent the survey organization from recontacting the intended respondent (Stokes and Jones 1989). Further indicators focus on answers given to specific types of survey questions (e.g., scales, filter questions). In general, falsifiers tend to produce lower response variance within and between interviews compared to honest interviewers (Schäfer et al. 2004; Menold et al. 2013). This is driven by a variety of strategies or behaviors. For instance, falsifiers rely on their preconceived opinions or group stereotypes to provide plausible answers that a particular respondent (e.g., student, homemaker, migrant) might provide during an interview (Reuband 1990). Falsifiers also have a tendency for choosing answers in the middle of ordinal response scales rather than extreme values to avoid suspicious inconsistencies (Porras and English 2004; Storfinger and Winker 2013). They tend to avoid item nonresponse by providing answers to all closed-ended questions (Bredl, Winker, and Kötschau 2012). To reduce implausible answer combinations, which could raise suspicion, falsifiers rarely show acquiescent response behavior, that is, the tendency to agree or answer “yes” to opinion items. To decrease their effort, falsifiers often choose answers that trigger fewer follow-up questions due to filtering (Hood and Bushery 1997; Eckman et al. 2014). Altogether, these behaviors lead to reduced variation in the data.

Furthermore, real respondents hear the questions, whereas falsifiers read and answer the questions as in a self-administered mode, which may lead to different primacy (choosing the first options of answer lists) and recency effects (choosing the last options of answer lists) (Menold et al. 2013). Respondents also show a higher rounding tendency in open numeric questions (e.g., income, working hours) compared to falsifiers (Menold et al. 2013). Additionally, falsifiers tend to avoid answering open-ended items, leading to higher rates of nonresponse and less frequent selection of the “Other, specify” option for semi-open-ended questions, which is contrary to other question types (Bredl, Winker, and Kötschau 2012). Benford’s Law is another example, which states that the first digit of naturally occurring numbers follows a logarithmic distribution (Benford 1938; Hill 1999). It is often utilized to evaluate the veracity of numeric data, since falsifiers are less likely to reproduce the Benford distribution (Schäfer et al. 2004).

*New falsification indicators:* In addition to the indicators from previous research described above, we propose four new falsification indicators: the rate of provided email addresses, a measure of the relative interview duration, the rate of respondent consent to link their survey data to administrative data, and the interviewers’ evaluation of their interviews. The rate of provided email addresses follows the same logic as the paradata indicator on telephone numbers: falsifiers tend to produce more missing email addresses to prevent the verification of the interview. Relative interview duration (average interview duration per question) is expected to be lower for falsifiers, as it reflects different types of time-saving behavior (e.g., avoidance of triggering follow-up questions to filter items, not reading/repeating questions out loud). Falsifiers are expected to produce higher linkage consent rates compared to real interviewers because granting linkage consent is viewed as a desirable research outcome and is an indication of cooperative response behavior that is unlikely to raise suspicion. Finally, given that falsifiers aim to produce inconspicuous and generally cooperative interviews in order to avoid detection, we expect falsifiers’ post-interview evaluation of the interview (i.e., the interviewer evaluation) to be very positive compared to those of honest interviewers.

## Data, Methods, and Evaluation Strategy

### Data

We utilize data from the IAB-BAMF-SOEP Survey of Refugees in Germany (Brücker, Rother, and Schupp 2017), including verified falsifications (version SOEP.v33) (Kosyakova et al. 2019).^1^ This is an annually conducted longitudinal household survey, launched in 2016. The target population includes refugees and asylum seekers who arrived between 2013 and 2016, and their adult household members.^2^ The sample was drawn from the German Central Register of Foreigners (Ausländerzentralregister) (Kroh et al. 2017). We use data of the first wave with a sample of 3,554 responding households and 4,816 respondents.^3^

The household-level response rate (Response Rate 2; AAPOR 2016) was 48.7 percent (Kroh et al. 2017). The survey included two types of questionnaires: person interviews, ideally conducted with every adult household member, and a shorter household interview with the anchorperson about the household’s situation. A staff of 98 trained interviewers, who worked in specific regional areas, completed between one and 289 (mean ≈ 49, median ≈ 32) computer-assisted personal interviews (CAPI). Interviewing started at the end of June 2016 and was completed in December 2016 (Brücker, Rother, and Schupp 2017). Since the sample included refugees from various home countries—in part without German language proficiency—questionnaires were provided in seven languages (Arabic, English, Farsi/Dari, German, Kurmanji, Pashtu, and Urdu). Additionally, the questionnaires were complemented with audio files containing recordings of the questions and access to an interpreter hotline (Jacobsen 2018). Person-level questionnaires included principal topics on migration history, education biographies, language acquisition and employment, life satisfaction, health, and attitudes (Brücker, Rother, and Schupp 2017).

Routine quality control checks by the survey organization detected a first suspicious interviewer, who was confirmed as a falsifier after a subsequent review of her wave 1 respondents (IAB 2017). We refer to this interviewer as “F1.” F1 accounted for 289 person interviews and 217 household interviews, which must be considered as complete falsifications. Further investigations carried out by the survey organization and the IAB (including various statistical methods, recontacting of respondents, and questioning of supervisors and interviewers) confirmed two additional falsifiers responsible for a total of 62 person and 47 household interviews (DIW 2019; Kosyakova et al. 2019). These interviewers did not fabricate all of their assigned interviews. According to the survey organization, only in the latter half of the field period did these interviewers start fabricating complete interviews. The exact number of these falsified interviews could not be determined and is unknown. We refer to these interviewers as “F2” and “F3.” Consistent with the AAPOR definition of interviewer falsification (AAPOR 2003), we refer to interviewers F1, F2, and F3 as falsifiers and the data produced by these interviewers as falsifications. Table 1 contains detailed information about the number of interviews (overall and for each falsifier) and response rates.

**Table 1.** Response outcomes for falsifiers and nonfalsifiers

		Person interviews		Household interviews	
	Response rate	*N*	Pct.	*N*	Pct.
Falsifier					
F1	85.8%	289	6.0%	218	6.1%
F2	60.7%	46	1.0%	34	1.0%
F3	41.9%	16	0.3%	13	0.4%
Total for falsifiers	77.7%	351	7.3%	265	7.5%
Total for nonfalsifiers	48.4%	4,465	92.7%	3,289	92.5%
Total	48.7%	4,816	100.0%	3,554	100.0%

Source *.—*IAB-BAMF-SOEP Survey of Refugees in Germany (version SOEP.v33).

Note.—All response rates are calculated at the household level according to Response Rate 2 (AAPOR 2016).

### Statistical Detection Methods

#### Cluster analysis

Starting with cluster analysis (see, e.g., Kaufman and Rousseeuw 1990), the basic idea is to classify interviewers into smaller homogeneous subgroups that distinguish suspicious and nonsuspicious interviewers using grouping characteristics (i.e., the falsification indicators). To evaluate the distances between interviewers, we implement the commonly used Euclidean distance:

(1) $$d_{j,l}={\left[\sum_{k=1}^{n}{\left(x_{jk}-x_{lk}\right)}^{2}\right]}^{\frac{1}{2}}$$

with $d_{j,l}$ denoting the distance between a pair of interviewers *j* and *l*, and $x_{jk}$ and $x_{lk}$ denoting the values for the *k*^th^ (*= 1, 2, …, n*) falsification indicator for the respective interviewer pair. Based on the resulting distance matrix, classification can take place using different clustering algorithms, which greatly differ with regard to the group formation. We compare two hierarchical-agglomerative algorithms: Ward’s Linkage (Ward 1963) and Single-Linkage (McQuitty 1957).

In the context of falsification identification, Ward’s Linkage has been successfully applied in previous research (Menold et al. 2013; Storfinger and Winker 2013). Ward’s Linkage combines clusters such that the sum of squared errors is minimized. This allows varying cluster sizes, which enables a meaningful cluster solution even for—as we assume—a small group of potential falsifiers. In contrast to Bredl, Winker, and Kötschau (2012) and Menold et al. (2013), we allow for solutions with more than two clusters. The rationale for permitting solutions with more than two groups is that the falsification indicators could also capture different interviewing styles and behaviors (e.g., differences between experienced and inexperienced interviewers) that may not be fraudulent in nature. Hence, greater separation of these interviewing styles is enabled, minimizing the risk of unwarranted suspicions against honest interviewers that might occur in a forced two-group solution. However, this approach impedes direct identification of the suspicious group and requires further inspection of each group based on a comparison of their indicator values. In contrast to prior studies, we additionally apply Single-Linkage to address the problem of identifying suspicious interviewers. Single-Linkage^4^ is particularly useful for identifying outliers, since it combines clusters that have the closest neighboring objects (Kaufman and Rousseeuw 1990).

To determine the optimal cluster solution for both Ward’s Linkage and Single-Linkage, we visually inspect dendrograms and further consider the formal criteria of the Calinski-Harabasz index and the Duda-Hart index (Caliński and Harabasz 1974; Duda and Hart 1973). Optimal cluster solutions are indicated by large values of the Calinski-Harabasz pseudo F-index and Duda-Hart Je(2)/Je(1)-index as well as small values of the Duda-Hart pseudo T-squared. First, we derive from the dendrogram which cluster solutions are plausible according to the shown dissimilarity measure. Second, we compare the values of the formal criteria for these cluster solutions.

#### Meta-indicator approach

As described above, the application of cluster analysis requires several decisions, which may affect the results. We therefore propose a simpler multivariate tool, which we refer to as the *meta-indicator approach*. Basically, it summarizes the interviewer-level values of all indicators into a single (meta-)indicator value per interviewer: First, to obtain comparable and continuous values for each of the indicators, each interviewer-level indicator value is standardized across all interviewers using the following equation:

(2) $$z_{i,k}=\frac{x_{i,k}-{\bar{x}}_{k}}{S_{k}}$$

with $z_{i,k}$ denoting the *k*^th^ standardized indicator value for interviewer *i* and $x_{i,k}$ denoting the unstandardized indicator value. Further, ${\bar{x}}_{k}$ denotes the mean value of indicator *k* and $S_{k}$ the corresponding standard deviation. Second, all standardized indicator values are summed up for each interviewer. Note that indicator values are coded such that positive values indicate the assumed suspicious direction. Therefore, extreme positive values of the meta-indicator signal potential falsification behavior of interviewers. We consider three arbitrary thresholds, which flag interviewers as “suspicious” if their meta-indicator value exceeds it to demonstrate the sensitivity of the method under more inclusive and restrictive identification criteria. The first threshold is defined as 2 standard deviations (SD) above the mean, which is a commonly used “rule-of-thumb” for outlier detection, especially in relatively small samples.^5^ The second and third thresholds are 1.75 and 2.25 SDs above the mean, which represent more liberal and conservative identification criteria, respectively, compared to the 2 SD rule. In practice, the actual threshold can be adapted flexibly, even after inspection of the overall distribution, depending on the user’s preference for a more inclusive or restrictive controlling process.

#### Falsification indicators

In total, we consider 32 falsification indicators: 21 based on person-level data (interview data, paradata, and interviewer’s evaluation of the person interview) and 11 on household-level data (interview data and paradata). All indicators are standardized according to equation (2) and coded such that positive values indicate the suspicious direction; for example, interviewers with a lower share of item nonresponse—the assumed direction of falsification for closed-ended items—receive a larger positive indicator value compared to interviewers with a higher share of item nonresponse. Further, the interview-level values of a falsification indicator were aggregated to the interviewer level by computing the mean indicator value across all interviews of an interviewer. Table 2 provides a summary of the used indicators, their assumed direction for falsifiers, and a description of their construction. Further information about the used indicators is shown in table 3. The actual observed indicator values, shown separately for falsifiers and honest interviewers, are given in Supplementary Material table 1. Note that we do not account for area-level effects, as this could hinder the identification of falsifiers collaborating in certain regions, as seen in Bergmann, Schuller, and Malter (2019). Likewise, we do not account for nonindependence within households, as all analyses are conducted at the interviewer level.

**Table 2.** Overview of used falsification indicators and underlying assumptions

Indicator	Description	Assumed direction of falsifiers	References
Acquiescent responding	Fraction of positive connotation (“Agree/Strongly Agree”) independent of content	Lower fraction of positive connotation independent of question content for falsifiers	Menold et al. (2013)
Benford’s Law	Decreasing distribution of leading digit for numeric quantities	Poor fit of Benford’s distribution to leading digits for falsifiers	Swanson, Cho, and Eltinge (2003)
Email	Fraction of email address provision	Lower fraction of provided email addresses for falsifiers	NEW
Extreme responses	Fraction of extreme responses to rating scales	Lower fraction of extreme responses to rating scales for falsifiers	Schäfer et al. (2004)
Filter questions	Fraction of responses which lead to follow-up questions	Lower fraction of responses which lead to follow-up questions for falsifiers	Hood and Bushery (1997)
Interview duration	Duration of completed interviews	Shorter duration of completed interviews for falsifiers	Hood and Bushery (1997)
Interview duration, relative	Duration of completed interviews relative to the triggered questions	Shorter duration of completed interviews relative to the triggered questions for falsifiers	NEW
Interviewer evaluation	Interviewer’s evaluation of the interview situation	Higher fraction of very positive evaluation of the interview situation for falsifiers	NEW
Item nonresponse	Item nonresponse rate within an interviewer’s workload of closed-ended questions	Lower item nonresponse rate for falsifiers	Schäfer et al. (2004)
Middle-category responses	Fraction of middle responses to rating scales	Higher fraction of middle responses to rating scales for falsifiers	Schäfer et al. (2004)
Nondifferentiation	Standard deviation within an item scale	Lower standard deviation within an item scale for falsifiers	Reuband (1990)
Primacy effects	Fraction of choosing the first two categories in nonordered answer option lists	Higher fraction of choosing the first two categories in nonordered answer option lists for falsifiers	Menold et al. (2013)
Recency effects	Fraction of choosing the last two categories in nonordered answer option lists	Lower fraction of choosing the last two categories in nonordered answer option lists for falsifiers	Menold et al. (2013)
Record linkage consent	Fraction of consent to record linkage	Higher fraction of consent to record linkage for falsifiers	NEW
Rounding	Fraction of rounding numbers in numerical open-ended questions	Lower fraction of rounded numbers in numerical open-ended questions for falsifiers	Menold et al. (2013)
Semi-open responses	Fraction of responses to “other” in semi-open-ended question	Lower fraction of responses to “other” in semi-open-ended question for falsifiers	Hood and Bushery (1997)
Stereotyping	Strength of stereotypical response to attitudinal items	Higher strength of stereotypical response to attitudinal items for falsifiers	Reuband (1990)
Telephone number	Fraction of telephone number provision	Lower fraction of provided telephone numbers for falsifiers	Stokes and Jones (1989)
Response variance	Standard deviation of responses between interviews	Lower standard deviation of responses between interviews for falsifiers	Porras and English (2004)

Source.—The table was adapted from Kosyakova et al. (2019).

**Table 3.** Overview of used falsification indicators and labels

Indicator	Data source	Label
Acquiescent responding	Person interviews	ACQ_P
Benford’s Law	Person interviews	BFL_P
	Household interviews	BFL_H
Email	Household-level paradata	MAIL_H
Extreme responses	Person interviews	ERS_P*
	Household interviews	ERS_H
Filter questions	Person interviews	FILT_P
	Household interviews	FILT_H
Interview duration	Person interviews	DUR_P
	Household interviews	DUR_H
Interview duration, relative	Person interviews	RDUR_P
	Household interviews	RDUR_H
Interviewer evaluation	Person-level evaluation	EVAL_P
Item nonresponse	Person interviews	INR_P
	Household interviews	INR_H
Middle-category responses	Person interviews	MRS_P*
	Household interviews	MRS_H
Nondifferentiation	Person interviews	ND_P
Primacy effects	Person interviews	PRIM_P
Recency effects	Person interviews	RECE_P
Record linkage consent	Person-level paradata	RLC_P
Rounding	Person interviews	ROUND_P
	Household interviews	ROUND_H
Semi-open responses	Person interviews	SOR_P
Stereotyping	Person interviews	STEREO_P
Telephone number	Household-level paradata	TEL_H
Response variance	Person interviews	VAR_P
	Household interviews	VAR_H

*Due to large differences in the number of scale categories between item batteries, three different indicators were created. Large scales with 10 or 11 answer categories (h), medium size scales with 7 categories (m), and small scales with 4 or 5 categories (l).

### Evaluation Strategy

#### Comparison of multivariate detection methods

To evaluate the performance of the different detection methods in identifying the falsifiers, we consider several quality measures: false-positive rate, false-negative rate, accuracy, error rate, and Cohen’s kappa (Cohen 1960). The false-positive rate relates the number of falsely detected interviewers to the overall number of honest interviewers, whereas the false-negative rate measures the share of overlooked falsifiers. The accuracy captures the relationship between the false-negative and false-positive rates, whereas the error rate equals one minus the accuracy. Cohen’s kappa adjusts the accuracy by accounting for the possibility of true predictions by chance. Corresponding formulas can be found in table 4. We test the robustness of the cluster analyses and the meta-indicator results by applying a simple leave-one-out procedure, repeating the respective analyses excluding one indicator at a time.

**Table 4.** Overview of formulas for performance measures

Performance measure	Formula	
False-positive rate (${FP}_{\mathrm{rate}}$)	FP/(TN+FP)	(3)
False-negative rate (${FN}_{\mathrm{rate}}$)	FN/(TP+FN)	(4)
Accuracy (A)	(TP+TN)/(TP+TN+FP+FN)	(5)
Error rate ($E_{\mathrm{rate}}$)	1-A	(6)
Cohen’s kappa (κ)	$\left({Pr(a)}_{\mathrm{obs}}-{\mathrm{Pr}\left(b\right)}_{\;\text{exp}\;}\right)/\left(1-{\mathrm{Pr}\left(b\right)}_{\;\text{exp}\;}\right)$	(7)

Note *.—FP* = false-positive cases, *FN* = false-negative cases, *TP* = true-positive cases, *TN* = true-negative cases, ${\mathrm{Pr}(a)}_{\mathrm{obs}}$ = observed agreement, ${\mathrm{Pr}\left(b\right)}_{\;\text{exp}\;}$ = expected agreement.

#### Comparison of single indicators

We use discriminant analysis to evaluate the relative importance of the single indicators for identifying falsifiers and to test the validity of the directional assumptions of the indicators (Bredl, Winker, and Kötschau 2012). Linear discriminant analysis is not used as an instrument to detect falsifiers but enables assessment of the goodness-of-falsification indicators in distinguishing falsifiers from the nonfalsifiers if falsifiers are known. Using a linear combination of the continuous standardized indicator variables $z_{k}$ (*k = *1, 2, …, *n*) as independent discriminating variables, we seek the canonical discriminant function that provides the maximal separation between the falsifier and nonfalsifier groups (Klecka 1980; McLachlan 2004). The discriminant function *D* takes the following form:

(8) $$D=b_{0}+b_{1}z_{1}+b_{2}z_{2}+\cdots +b_{n}z_{n}=b_{0}+\sum_{k=1}^{n}b_{k}z_{k}$$

Due to the binary falsification status, only one discriminant function is determined. Maximal discrimination is achieved by determining the discriminant constant $b_{0}$ and the discriminant coefficients $b_{k}$ such that the group-specific $D_{g}=1/I_{g}\sum_{i=1}^{I_{g}}D_{ig}$—with g=1 for falsifiers, g=0 for honest interviewers, and $I_{g}$ the number of interviewers per group—are as different as possible (Klecka 1980; Bredl, Winker, and Kötschau 2012). Put differently, the aim is to maximize the between-group variance but minimize the within-group variance. The absolute sizes of the standardized discriminant coefficients identify the most important indicators for the distinction between falsifiers and nonfalsifiers. Since some of the indicators are highly correlated, we consider the canonical structure coefficients, which adjust for possible multicollinearity between indicators. Note that a comparison of the standardized coefficients and the structure coefficients deepens the understanding of the underlying relationships between the indicators and allows for assessing the importance of single indicators.

## Results

### Cluster Analysis

**Figure 1.**
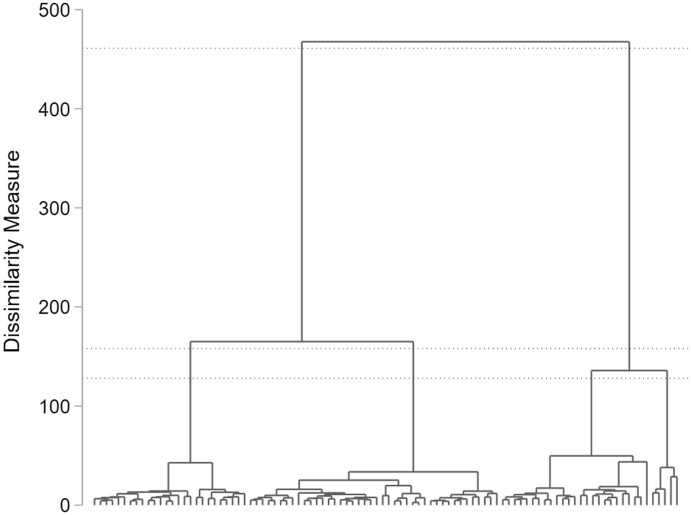
**Full dendrogram for Ward’s Linkage cluster analysis.** Dotted lines indicate plausible cluster solutions. Source.—IAB-BAMF-SOEP Survey of Refugees in Germany (version SOEP.v33).

*Ward’s Linkage*: Figure 1 shows the full dendrogram according to the dissimilarity between the groups for Ward’s Linkage. The dotted lines indicate plausible cluster solutions. Accordingly, a 2-, 3-, or 4-cluster solution seems plausible.

**Figure 2.**
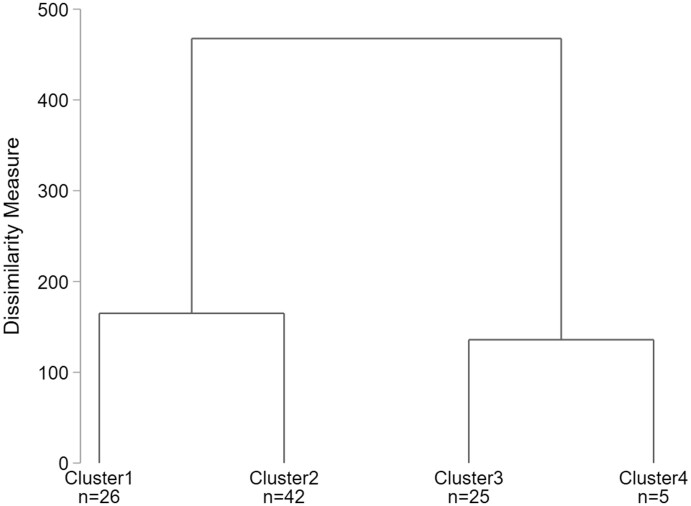
**Dendrogram for Ward’s Linkage cluster analysis with 4-cluster solution.** Source.—IAB-BAMF-SOEP Survey of Refugees in Germany (version SOEP.v33).

Looking at the two formal indices (table 5), we find contrary recommendations: Calinski-Harabasz suggests a 2-cluster solution, whereas Duda-Hart suggests a 4-cluster solution. Looking at the number of interviewers per cluster, the 4-cluster solution with 26, 42, 25, and 5 interviewers rather than 68 and 30 interviews seems more plausible, since we assume falsifiers to be the minority among interviewers. The dendrogram for the 4-cluster solution is presented in figure 2.

**Table 5.** Calinski-Harabasz and Duda-Hart Index for Ward’s Linkage and Single-Linkage

Number of clusters	Calinski-Harabasz	Duda-Hart	
	Pseudo F-index	Je (2)/Je (1) index	Pseudo T-squared
Ward’s Linkage			
1	–	0.515	90.27
2	90.27	0.587	46.35
3	64.21	0.516	26.24
**4**	**79.93**	**0.777**	**6.60**
5	66.45	0.477	10.96
6	62.08	0.691	10.74
7	56.51	0.506	2.93
8	56.44	0.839	7.69
9	52.92	0.000	–
10	52.91	0.828	5.84
Single-Linkage			
1	–	0.959	4.07
2	4.07	0.886	12.19
3	8.37	0.833	18.81
4	12.89	0.788	25.02
**5**	**18.40**	**0.365**	**1.74**
6	14.90	0.000	–
7	12.44	0.962	3.59
8	11.48	0.968	2.91
9	10.62	0.969	2.85
10	9.96	0.987	1.16

Source.—IAB-BAMF-SOEP Survey of Refugees in Germany (version SOEP.v33).

Note.—The chosen cluster solution used for the evaluation is indicated in boldface.

**Figure 3.**
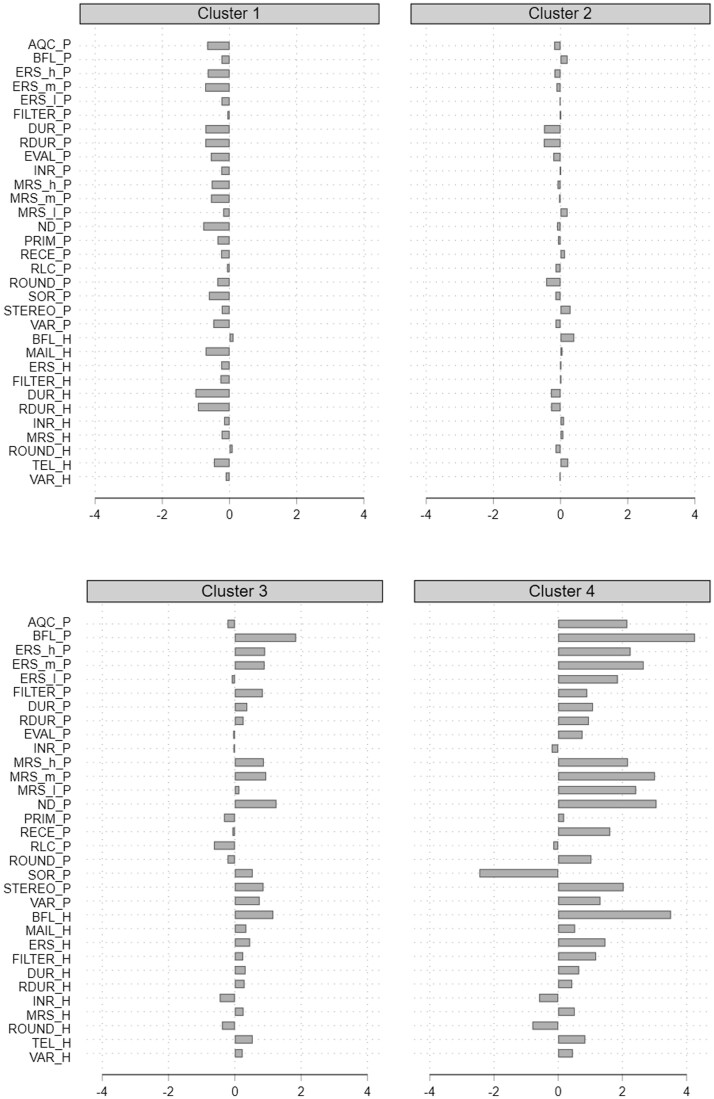
**Mean indicator values per cluster for Ward’s Linkage.** Source.*—*IAB-BAMF-SOEP Survey of Refugees in Germany (version SOEP.v33).

To identify the suspicious group, inspection of the mean indicator values for each cluster is necessary. The results in figure 3 imply that Cluster 1 mostly includes interviewers with negative indicator values, while Cluster 2 mainly includes interviewers with indicator values around zero. Both groups are therefore associated with unsuspicious interviewer behavior. Cluster 3 includes interviewers with mixed indicator values, having a slight tendency for suspicious values. In practice, one might consider randomly sampling some interviews for reinterviews from this group of interviewers. More severe is Cluster 4, which includes interviewers with highly suspicious indicator values for most indicators. This group clearly stands out as being suspicious, compared to the other groups, and would be a prime target for further investigation via reinterviews.

Subsequent inspection revealed that the outlying cluster includes all three falsifiers (F1, F2, and F3) but also two further interviewers (I62 and I70). However, since these two interviewers conducted a very small number of (i.e., less than five) interviews, the indicator values could reflect respondents’ answering behavior rather than deviant interviewing. Controls conducted by the survey organization did not confirm any suspicious behavior for these two interviewers.

Table 6 shows the false-positive rates, false-negative rates, as well as the accuracy, error rate, and kappa statistic for the different detection methods. For the 4-cluster solution, Ward’s Linkage (first column) results in a false-positive rate of 2.1 percent and a false-negative rate of 0 percent. Because of the low false-positive and false-negative rates, accuracy is very high (98.0 percent) and the error rate very low (2.0 percent), also resulting in a very good kappa statistic (0.74).

**Table 6.** Performance measures of interviewer falsification detection methods

	Ward’s Linkage	Single-Linkage	Meta-indicator thresholds		
			1.75 SDs	2.00 SDs	2.25 SDs
False-positive rate	2.1%	3.2%	2.1%	2.1%	2.1%
False-negative rate	0.0%	0.0%	0.0%	0.0%	40.0%
Accuracy rate	98.0%	97.0%	98.0%	98.0%	95.9%
Error rate	2.0%	3.1%	2.0%	2.0%	4.1%
Kappa statistic	0.74	0.65	0.74	0.74	0.31

Source.—IAB-BAMF-SOEP Survey of Refugees in Germany (version SOEP.v33).

**Figure 4.**
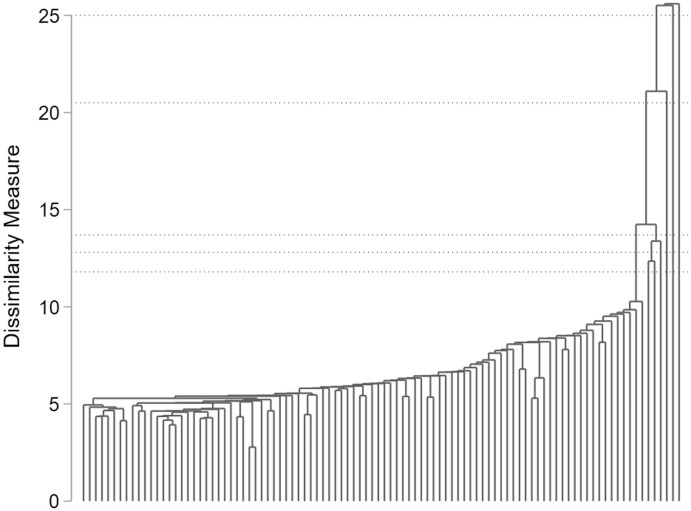
**Full dendrogram for Single-Linkage cluster analysis.** Dotted lines indicate plausible cluster solutions. Source.— IAB-BAMF-SOEP Survey of Refugees in Germany (version SOEP.v33).

*Single-Linkage:* Figure 4 shows the full dendrogram according to the dissimilarity measurement for Single-Linkage. As for Ward’s Linkage, dotted lines indicate plausible cluster solutions, ranging between three and seven clusters. The figure further indicates that most interviewers (in total 92) share a high similarity, whereas six interviewers appear as outliers and therefore as suspicious.

Considering the formal indices (table 6), we find great support for a 5-cluster solution according to the Calinski-Harabasz index. The recommendation of the Duda-Hart index is ambiguous: the Pseudo T-squared of the index also supports the 5-cluster solution, whereas the Je(2)/Je(1)-index supports a 7-cluster solution. The decision between the two solutions is arbitrary, as both identify the same outliers, with three outliers grouped together in the 5-cluster solution and placed in separate clusters in the 7-cluster solution.

**Figure 5.**
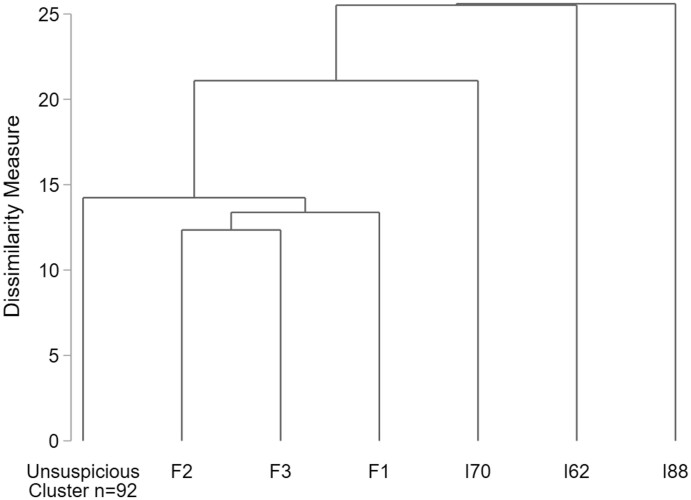
**Dendrogram for Single-Linkage cluster analysis with 7-cluster solution.** Source.—IAB-BAMF-SOEP Survey of Refugees in Germany (version SOEP.v33).

The dendrogram for the 7-cluster solution (figure 5) reveals that, similar to Ward’s Linkage, all three falsifiers (F1, F2, and F3) are identified as suspicious. Three further interviewers characterized by a small number of conducted interviews (I62, I70, and I88) are falsely suspected. The falsifiers seem to be more similar than the other outlying interviewers, since they would be grouped together in a 5-cluster solution. Since the number of falsely suspected interviewers is slightly higher for Single-Linkage, accuracy, error rate, and kappa statistic result in a worse evaluation (table 6, second column).

### Meta-Indicator Approach

**Figure 6.**
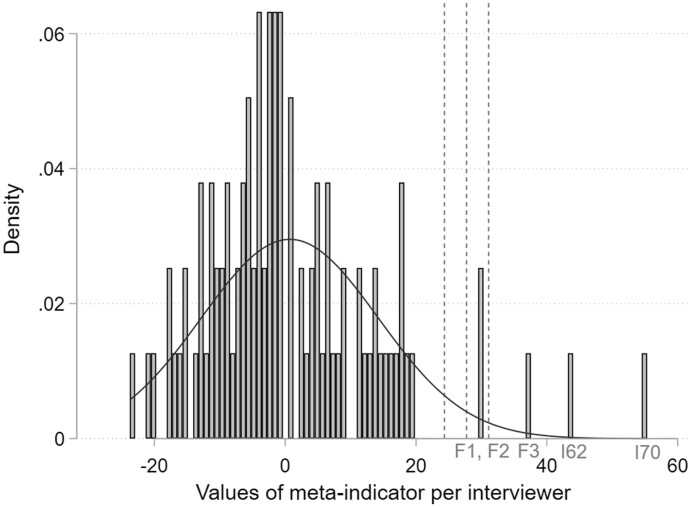
**Distribution of the meta-indicator values.** Dotted lines indicate the 1.75, 2.00, and 2.25 SD thresholds, respectively. Source*.—*IAB-BAMF-SOEP Survey of Refugees in Germany (version SOEP.v33).

Following our assumptions, the meta-indicator should produce extreme positive values for suspicious interviewers relative to the honest interviewers. As figure 6 shows, five outlying interviewers (including all falsifiers and two further interviewers) lie above the predefined threshold values of 1.75 and 2 SDs above the mean; I62 and I70 are again falsely suspected. This is also confirmed using a boxplot, which can be found in the Supplementary Material, figure 1.

Similar to Ward’s Linkage, both meta-indicator thresholds (1.75 and 2 SDs) result in a false-positive rate of 2.11 percent and a false-negative rate of zero percent and therefore the same accuracy, error rate, and kappa statistic (table 6, third and fourth columns). However, the more conservative threshold of 2.25 SDs above the mean results in poorer performance. Two falsifiers (F1 and F2) would be classified as unsuspicious, resulting in a high false-negative rate of 40 percent (table 6, fifth column). This slightly affects the accuracy (95.9 percent) and the error rate (4.1 percent). However, the kappa statistic drops drastically from 0.74 to 0.31.

### Sensitivity of Detection Methods by Indicator

*Cluster Analysis*: Repeating the analysis of the different cluster algorithms with a leave-one-out procedure for each indicator reveals very stable results. Regardless of which indicator is left out, both Single-Linkage and Ward’s Linkage consistently identified all three falsifiers. All falsely suspected interviewers are also identified as suspicious. Accordingly, the false-positive and false-negative rate and therefore also the other performance measures do not change.

*Meta-Indicator*: For the meta-indicator, the results are mostly stable, depending on the selected threshold. Regardless of the indicator left out, all falsifiers are clearly identified as suspicious using the 1.75 SD threshold. Importantly, this does not increase the number of falsely suspected interviewers. The more conservative 2 SD threshold leads to a slightly worse performance. F3 is always identified as suspicious; however, F1 and F2 are not identified in all cases. Particularly, F1 is overlooked if the indicator for primacy effects (PRIM_P), interviewer evaluation (EVAL_P), or rounding tendency (ROUND_P, ROUND_H) is left out. F2 is not flagged if the indicator for semi-open responses (SOR_P), Benford’s Law (BFL_P), nondifferentiation (ND_P), or middle-responding-style (MRS_h_P, MRS_m_P) is left out. This is reinforced by using the most conservative threshold of 2.25 SDs. Again, F3, I62, and I70 remain in the suspicious group regardless of the withdrawn indicator. F1 is labeled as suspicious for only five (out of 32) versions of the reduced meta-indicator, and F2 for only nine versions of the reduced meta-indicator.

### Comparison of Single Indicators Using Discriminant Analysis

To assess the relative importance of the single indicators, we turn to the discriminant analysis. The canonical correlation—which is equivalent to the Pearson correlation between the falsification status and the best linear combination of all indicators—is 0.757 (table 7). Hence, the combination of indicators is highly correlated with the actual falsification status. This is also confirmed by Wilks’s lambda (significant at an alpha level of 0.000).

**Table 7.** Model-fit of the discriminant analyses

	Canonical Correlation	Eigenvalue	Wilks’ Lambda	F	df1	df2	*p*-value
Function D	0.757	1.346	0.426	2.734	32	65	0.000

Source.—IAB-BAMF-SOEP Survey of Refugees in Germany (version SOEP.v33).

Note.—*P*-values are based on a one-tailed significance test.

However, there are remarkable differences between the relative importance of single indicators for the used data. The resulting group-specific discriminant value $D_{g}$ for the falsifier group (g=1) amounts to −6.461 and 0.204 for the nonfalsifier group (g=0). Accordingly, the group of falsifiers is associated with negative values on the canonical variables. This is important for the interpretation of the coefficients, given that negative coefficients indicate conformity of the directional assumptions of the indicators. Table 8 presents the standardized discriminant coefficients as well as the canonical structure coefficients for all 32 indicators. The absolute magnitude of the standardized coefficients infers on the importance of the single indicators for the discrimination between falsifiers and nonfalsifiers in a joint model of all 32 indicators. The person-level interview duration indicator (DUR_P) and the newly developed relative counterpart (RDUR_P) seem to be of utmost importance. However, due to their significant correlation (Supplementary Material, table 2), the coefficient for duration is negative whereas the coefficient for relative duration is positive, since the effect of the relative duration is already captured by the duration indicator. Hence, it would probably suffice to use only one of these indicators in practice. Further, the number of triggered filter questions in person interviews (FILT_P) and the relative duration of the household interview (RDUR_H) are also crucial. All four indicators are related measures, highlighting the importance of time-related measures or measures indicating potential shortcutting for detecting falsifiers.

**Table 8.** Results of the discriminant analysis

Indicator	Interview type	Standardized discriminant coefficients	Ranking	Canonical structure coefficients	Ranking
DUR_P	person level	−12.030	1	−0.205	6
*RDUR_P*	person level	10.608	2	−0.204	7
FILT_P	person level	2.630	3	−0.035	27
*RDUR_H*	household level	0.962	4	0.033	28
BFL_P	person level	−0.674	5	−0.155	14
MRS_h_P*	person level	−0.605	6	−0.330	2
ND_P	person level	−0.506	7	−0.327	3
ERS_m_P*	person level	0.484	8	−0.201	8
ROUND_P	person level	−0.462	9	−0.089	21
FILT_H	household level	−0.400	10	−0.080	24
ACQ_P	person level	−0.396	11	−0.161	13
MRS_H	household level	−0.392	12	−0.094	20
*EVAL_P*	person level	−0.384	13	−0.274	4
MRS_m_P*	person level	−0.351	14	−0.367	1
ERS_h_P*	person level	0.327	15	−0.194	10
DUR_H	household level	−0.313	16	0.003	32
PRIM_P	person level	−0.293	17	−0.179	12
VAR_H	household level	−0.282	18	−0.020	29
MRS_l_P*	person level	0.267	19	−0.049	26
*RLC_P*	person level	−0.241	20	−0.109	18
STEREO_P	person level	0.187	21	−0.192	11
INR_P	person level	0.162	22	−0.007	31
VAR_P	person level	0.146	23	−0.142	15
*MAIL_H*	household level	0.114	24	−0.077	25
ROUND_H	household level	0.091	25	0.109	17
SOR_P	person level	0.090	26	−0.084	22
ERS_l_P*	person level	−0.082	27	−0.114	16
TEL_H	household level	−0.060	28	−0.081	23
RECE_P	person level	−0.055	29	−0.197	9
BFL_H	household level	−0.041	30	0.014	30
ERS_H	household level	−0.030	31	−0.231	5
INR_H	household level	−0.013	32	0.100	19

Source.—IAB-BAMF-SOEP Survey of Refugees in Germany (version SOEP.v33).

*Due to large differences in the number of scale categories, three different indicators were created. Large scales with 10 or 11 answer categories (h), medium size scales with 7 categories (m), and small scales with 4 or 5 categories (l). New indicators are shown in italics.

We further observe that Benford’s Law at the person level (BFL_P) is central for the discrimination between falsifiers and nonfalsifiers. Another group of indicators plays an essential role: middle-responding-style (MRS_h_P), extreme-responding-style (ERS_m_P), and nondifferentiation (ND_P) in person interviews. Again, these indicators are correlated, since less extreme values automatically lead to more middle-category responses and therefore to more straightlining. Hence, ERS takes a positive value since the effect is already captured by MRS and ND. This demonstrates that item batteries serve as a crucial basis for falsification indicators. Turning to the newly proposed indicators, we find that, in addition to the relative duration, the interviewer’s evaluation (EVAL_P) of the interview serves as a valuable indicator. Although the indicator on record linkage consent (RLC_P) is inferior compared to the other new indicators, it is still useful in discriminating between falsifiers and nonfalsifiers and outperforms more than one-third of all indicators. The same is true for the number of provided email addresses (MAIL_H), which turned out to be of less importance relative to others, but still aided in the separation between the two groups.

To infer on the impact of single indicators without the influence of the other indicators, canonical structure coefficients—measuring the correlation between each indicator and the discriminant function—and their importance ranking are presented (table 8). These coefficients allow for testing the assumptions on the expected direction of the indicators (from table 2). As the falsifier group is associated with negative function values, negative values of the canonical structure reveal that an indicator points in the assumed direction of suspicion. Again, very low values do not contribute much to the explanation and are of lower importance. A total of 27 (out of 32) indicators, including all new indicators, point in the assumed direction of suspicion. All of the 21 person-level indicators are consistent with the assumptions regarding their direction. In turn, five household-level indicators are not in the assumed direction: Benford’s Law (BFL_H), interview duration (DUR_H), relative duration (RDUR_H), item nonresponse (INR_H), and rounding tendency (ROUND_H). With the exception of ERS_H, most household-level indicators have very low coefficient values. It is important to note that some indicators (e.g., interview duration [DUR], rounding tendency [ROUND], and item nonresponse [INR]) were generated for both interview types but resulted in contrary outcomes. Compared to the person-level interview, the household-level interview was much shorter with correspondingly fewer variables collected. Hence, indicators generated from a smaller set of variables might be characterized by lower explanatory power. Furthermore, answers to the household interview items were more homogeneous due to the special population,^6^ which may have limited the variation of these indicator values. Another possible explanation is the way in which the household data could be fabricated by the interviewers. Some household-related information might have been quite obvious for the falsifiers (e.g., composition, income, and accommodation type) or they might have conversed with the anchorperson but without a proper interview. This could have increased the “quality” of the household-level falsification and decreased the power of the indicators.

## Discussion

Even though statistical falsification detection methods can be powerful tools for improving the quality control process, comparative evaluations of different methods performed on real-world data are rare. We addressed this research gap by using large-scale survey data with verified falsifications and evaluated the performance of different multivariate detection methods (Ward’s Linkage clustering, Single-Linkage clustering, and the newly proposed meta-indicator) and numerous falsification indicators. Consistent with the literature (Menold et al. 2013; de Haas and Winker 2016), the results revealed pronounced effectiveness of the different multivariate detection methods utilizing various indicators in identifying all three confirmed falsifiers. Ward’s Linkage and the meta-indicator produced mostly the same accuracy, which was slightly higher than for Single-Linkage. By assessing the relative importance of single falsification indicators, we found—consistent with the literature (Hood and Bushery 1997; Li et al. 2011)—that time-related indicators are of crucial importance. This supports the notion that falsifiers aim to reduce their time investment when falsifying data. Furthermore, falsifiers failed in reproducing the Benford Distribution and were less successful in manipulating item batteries (Schäfer et al. 2004; Bredl, Winker, and Kötschau 2012; Menold et al. 2013). However, the importance of the indicators was sensitive to the level of interview data used to generate them. Indicators derived from person-level data were always in line with the directional assumptions and therefore proved to be of higher importance than those derived from household-level data.

### Practical Implications of Results

What do these results imply for practitioners? First, while both cluster analysis and the meta-indicator performed similarly well, the meta-indicator approach proved to be more straightforward and produced less ambiguous results. Therefore, the meta-indicator might be preferred for an initial screening of the data. We recommend that users visually inspect the meta-indicator distribution and use a lenient threshold to minimize the risk of overlooking falsifiers. Given the novelty of the approach, we encourage further applications and evaluations in other datasets to assess the generalizability of its performance and suitable thresholds. For a more thorough quality control, we recommend using both cluster analysis and the meta-indicator and compare their results. Note that statistical methods should be used in conjunction with routine nonstatistical approaches (e.g., reinterviewing) for better targeting and more efficient use of resources for catching falsifiers, but also for confirming suspected falsifiers identified by the statistical methods. This is important, as the premature removal of suspected falsified data without nonstatistical confirmation could lead to serious bias.

Second, the relative importance of the time-related indicators (e.g., interview duration), item scale indicators (e.g., middle-responding style), and record linkage consent was particularly high. Thus, we recommend incorporating them into statistical detection methods. However, almost all falsification indicators pointed in the direction of falsification behavior and indeed proved to be essential for identifying falsifiers, even though household-level indicators were less important than person-level ones. Since some falsifiers scored very low on certain indicators while others scored very high, considering as many indicators as possible is a good strategy to identify falsifiers.

### Limitations and Future Work

Although we showed that different detection methods performed similarly well in detecting falsifiers, each method has its drawbacks. While cluster analysis allows for identifying different interviewer groups that may reflect different interviewer behavior, it does require some technical decision-making regarding clustering algorithms and may still lead to ambiguous cluster solutions requiring further inspection and expert judgment. Furthermore, cluster analysis might not work as demonstrated if most of the interviewers are falsifiers. This also applies to the meta-indicator, which—while practically simple to implement—may also become difficult to interpret if the size of the interviewer staff is small.

We acknowledge that the results are based on a single dataset and data collection could be subject to specific opportunities and motives for the interviewers to falsify (Kosyakova et al. 2021). Hence, while the results are encouraging, these methods could work out differently for other datasets. Further, it is possible that the types of respondents assigned to an interviewer or the areas they worked in affected the results. However, such effects are unlikely for two reasons. First, due to the large number of indicators aggregated to the interviewer level, it is improbable that an honest interviewer is flagged solely on the type or behavior of their respondents (with the exception of interviewers with very few interviews). Second, upon their arrival, refugees were distributed exogenously according to national dispersal policies, which reduces the potential for area effects. We further acknowledge that most falsifications in the used data were complete falsifications, which are easier to detect than partial falsifications (DeMatteis et al. 2020). Evaluating detection methods for partial falsification is a topic for future work. Further, the statistical methods were applied only at the end of the field period. Although the demonstrated methods could be applied in “real time” during the field period, we are unable to assert how effective this would be. We encourage future studies to investigate this issue further. Future work should also consider the use of modern machine-learning methods (e.g., random forests, generalized boosted models), which could provide additional insights on the importance of indicators and their correlations.

## Data Availability Statement

REPLICATION DATA AND DOCUMENTATION are available at: https://osf.io/98wnt/. Data access was provided via researcher contacts at the Institute for Employment Research (IAB). External researchers may apply for access to these data by submitting a user-contract application to the SOEP Research Data Center (https://www.diw.de/en/diw_02.c.222836.en/data_access_and_order.html).

## Supplementary Material

SUPPLEMENTARY MATERIAL may be found in the online version of this article: https://doi.org/10.1093/poq/nfab066.

## Supplementary Material

nfab066_Supplementary_Data

## Footnotes

1 For the analyses, we are using version SOEP.v33. All falsifications were excluded from the official data release (v34).

2 Upon their arrival, refugees were distributed across Germany through a national dispersal allocation scheme (Königstein Key; Grote 2018).

3 All analyses are based on the raw field data; therefore, no weights are used and no design effects are considered.

4 Note that Single-Linkage is prone to chaining effects, that is, an interviewer might be added to a cluster because of a high similarity with a single interviewer within the cluster, even though the added interviewer shows high dissimilarity with the other interviewers in the cluster (Everitt and Rabe-Hesketh, 2006). However, in this particular application such effects are desirable, since we assume falsifiers to be strong outliers whereas honest interviewers may share some similarities but are still different from each other in other ways.

5 As further evaluation criteria, we considered the Interquartile Range (IQR), Tukey’s Method, the Median Absolute Deviation (MAD) Method, the Z-Score, and the Modified Z-Score. We did not find strong differences in the results between these methods and the SD-Method. Hence, we only present results for the SD-Method.

6 Roughly 50 percent of refugees resided in shared accommodations, which are likely to be similar to each other (Brücker, Kosyakova, and Vallizadeh 2020). Given that most of the surveyed refugees arrived in 2015 and 2016, approximately one year or less before the interview, their households were likely less heterogeneous than had they resided in Germany for 5–10 years.

## Notes

The authors thank Lukas Olbrich for providing feedback on earlier versions of this paper, Simon Huber and the KANTAR Team for providing additional information on the data collection process, and all those who commented on earlier presentations of this work at the following events: “Interviewers and Their Effects from a Total Survey Error Perspective” workshop in Lincoln, Nebraska, USA (February 2019); the 8^th^ Conference of the European Survey Research Association in Zagreb, Croatia (July 2019); and the 75^th^ Annual Conference of the American Association for Public Opinion Research (virtually, June 2020). Furthermore, the authors thank the editors and three anonymous reviewers for their helpful comments and suggestions. This work was supported by the joint doctoral program of the IAB and the University of Erlangen-Nuremberg (GradAB) [to S.S.] and the Charles Cannell Fund in Survey Methodology [to Y.K.].
