# Supplementary material for: How to Catch a Falsifier: Comparison of Statistical Detection Methods for Interviewer Falsification
Source: Public Opin Q. 2022 Feb 15;86(1):51–81. doi: 10.1093/poq/nfab066 (PMC8944307; doi:10.1093/poq/nfab066)
Supplement: nfab066_Supplementary_Data [file nfab066_supplementary_data.pdf]

How to Catch a Falsifier:

Comparison of Statistical Detection Methods for Interviewer Falsification

Silvia Schwanhäuser (corresponding author): Institute for Employment Research (IAB), Regensburger Str. 104, DE-90478 Nuremberg. Phone: +49 911 179 2770, E-Mail for correspondence: Silvia.Schwanhaeuser2@iab.de

Joseph W. Sakshaug: Institute for Employment Research (IAB), Regensburger Str. 104, DE-90478 Nuremberg. Phone: +49 911 179 9549, E-Mail: joe.sakshaug@iab.de

Yuliya Kosyakova: Institute for Employment Research (IAB), Regensburger Str. 104, DE-90478 Nuremberg. Phone: +49 911 179 3643, E-Mail: Yuliya.Kosyakova@iab.de

Main Sections of Supplementary Material:

- Supplementary Figures
- Supplementary Tables

Supplementary Material

SUPPLEMENTARY FIGURES

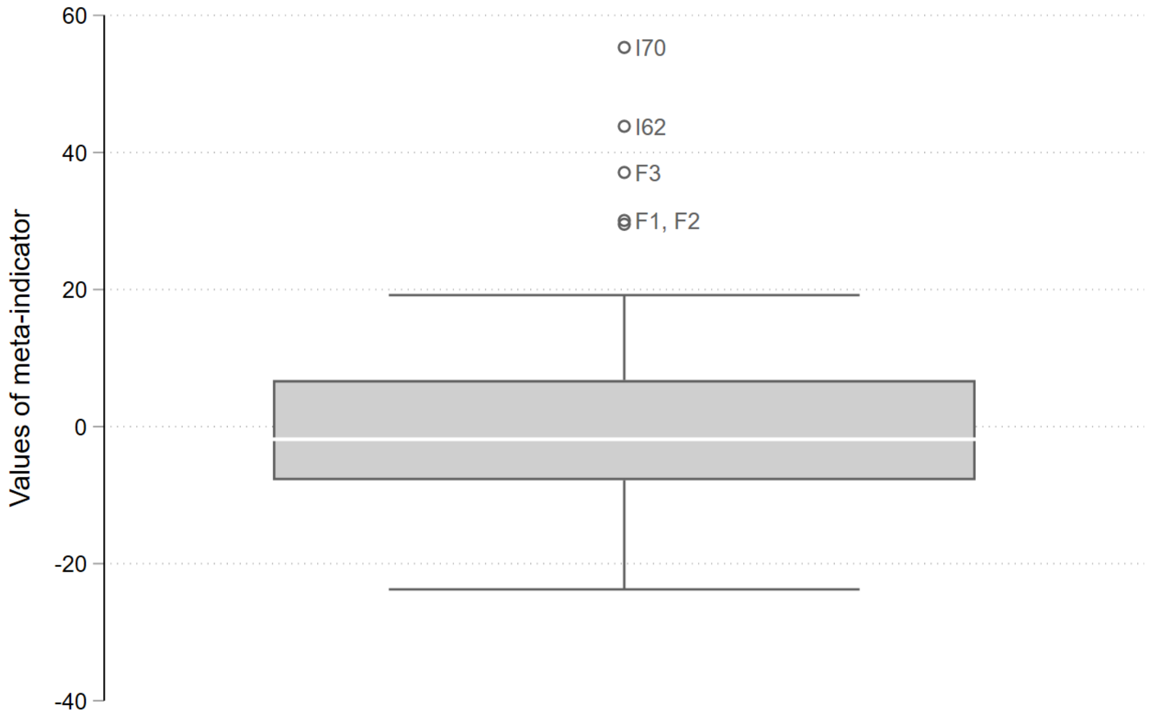

FIGURE S1. Boxplot of the meta-indicator values.

Source: IAB-BAMF-SOEP Survey of Refugees in Germany (version SOEP.v33).

SUPPLEMENTARY TABLES

Table S1. Interviewer-level indicator values for falsifiers and honest interviewers.

Indicator	Interview type	Total sample (excluding: F1, F2, and F3)	Falsifiers		
			F1	F2	F3
ACQ_P	person level	- 0.2034	0.8292	0.7016	2.6225
BFL_P	person level	0.7150	- 0.0306	6.5729	4.1294
BFL_H	household level	0.6794	0.1275	0.8487	0.5649
MAIL_H	household level	- 0.0505	0.5243	0.5243	0.5243
ERS_l_P*	person level	- 0.0045	2.1464	- 1.0774	1.6614
ERS_m_P*	person level	0.1209	0.4889	2.5633	2.4227
ERS_h_P*	person level	0.0979	0.3935	2.4313	2.0076
ERS_H*	household level	0.1312	0.9695	1.9803	2.2602
FILT_P	person level	0.2408	- 2.8745	2.8413	1.3776
FILT_H	household level	0.0575	- 0.2697	1.8224	0.6541
DUR_P	person level	- 0.2492	1.1268	0.9364	1.2291
DUR_H	household level	- 0.2765	0.7292	- 1.9405	0.3060
RDUR_P	person level	- 0.2881	1.4631	0.4753	1.0810
RDUR_H	household level	- 0.2733	0.7314	- 2.5821	0.2031
EVAL_P	person level	- 0.2136	2.7092	0.5304	1.2038
INR_P	person level	- 0.0883	1.4933	0.1866	- 1.7981
INR_H	household level	- 0.1463	0.5096	- 1.0332	- 2.2182
MRS_l_P*	person level	0.1988	2.1032	- 1.7229	1.5040
MRS_m_P*	person level	0.2318	0.4575	5.6283	2.8317
MRS_h_P*	person level	0.1554	- 0.8409	5.5442	2.9924
MRS_H	household level	0.0649	0.9029	1.3673	0.3939
ND_P	person level	0.2260	0.4544	5.2077	3.4337
PRIM_P	person level	- 0.2020	2.5178	- 0.6834	1.2532
RECE_P	person level	0.0543	2.3236	- 0.0324	1.9857
RLC_P	person level	- 0.2528	0.8336	0.8783	0.0715
ROUND_P	person level	- 0.2824	2.1264	- 2.6465	1.3690
ROUND_H	household level	- 0.1802	1.3899	- 1.8232	- 2.4090
SOR_P	person level	- 0.2154	0.4853	1.6854	0.9337
STEREO_P	person level	0.3892	1.9909	1.8848	1.7101
TEL_H	household level	0.1548	1.0279	0.5217	0.4511
VAR_P	person level	0.0661	2.5035	- 0.3120	0.7168
VAR_H	household level	0.0396	0.1848	- 1.2022	1.6111

Note: *Due to large differences in the number of scale categories, three different indicators were created. Large scales with 10 or 11 answer categories (h), medium size scales with 7 categories (m), and small scales with 4 or 5 categories (l). New indicators are shown in italics.
Source: IAB-BAMF-SOEP Survey of Refugees in Germany (version SOEP.v33).

Table S2. Correlations between falsification indicators.

	ACQ_P	BFL_P	ERS_h_P	ERS_m_P	ERS_l_P	FILT_P	DUR_P	RDUR_P	EVAL_P	INR_P
ACQ _P	1									
BFL_P	**- 0.41 (0.000)**	1								
ERS_h_P	**0.43 (0.000)**	0.01 (0.907)	1							
ERS_m_P	**0.55 (0.000)**	- 0.03 (0.765)	**0.90 (0.000)**	1						
ERS_l_P	**0.22 (0.031)**	- 0.19 (0.067)	**0.34 (0.001)**	**0.31 (0.002)**	1					
FILT_P	- 0.03 (0.746)	**0.46 (0.000)**	0.17 (0.102)	0.14 (0.169)	- 0.12 (0.245)	1				
DUR_P	0.11 (0.291)	**0.21 (0.038)**	0.13 (0.212)	0.19 (0.068)	- 0.14 (0.175)	**0.50 (0.000)**	1			
RDUR_P	0.12 (0.233)	0.16 (0.112)	0.13 (0.234)	0.19 (0.068)	- 0.14 (0.186)	**0.37 (0.000)**	**0.99 (0.000)**	1		
EVAL_P	0.13 (0.207)	- 0.06 (0.562)	0.02 (0.829)	0.04 (0.681)	0.07 (0.470)	- 0.02 (0.887)	0.20 (0.056)	**0.21 (0.042)**	1	
INR_P	**- 0.30 (0.003)**	- 0.12 (0.240)	- 0.15 (0.140)	- 0.17 (0.105)	0.03 (0.768)	- 0.30 (0.335)	- 0.11 (0.283)	- 0.07 (0.477)	0.07 (0.469)	1
MRS_h_P	0.18 (0.083)	0.07 (0.502)	**0.66 (0.000)**	**0.57 (0.000)**	**0.26 (0.011)**	**0.31 (0.002)**	0.01 (0.897)	- 0.02 (0.822)	- 0.03 (0.753)	0.03 (0.772)
MRS_m_P	0.25 (0.014)	0.10 (0.310)	**0.66 (0.000)**	**0.72 (0.000)**	**0.22 (0.033)**	**0.29 (0.004)**	0.13 (0.192)	0.11 (0.289)	0.10 (0.356)	0.19 (0.058)
MRS_l_P	- 0.07 (0.499)	0.18 (0.077)	0.16 (0.120)	0.14 (0.163)	**0.54 (0.000)**	- 0.04 (0.676)	- 0.00 (0.992)	0.01 (0.962)	- 0.04 (0.734)	0.18 (0.081)
ND_P	- 0.02 (0.847)	**0.46 (0.000)**	**0.61 (0.000)**	**0.64 (0.000)**	0.16 (0.124)	**0.48 (0.000)**	**0.32 (0.002)**	**0.28 (0.006)**	- 0.02 (0.864)	- 0.10 (0.341)
PRIM_P	**0.20 (0.049)**	**- 0.48 (0.000)**	0.07 (0.514)	0.09 (0.366)	0.12 (0.236)	**- 0.31 (0.002)**	- 0.09 (0.396)	- 0.05 (0.657)	- 0.16 (0.111)	0.06 (0.569)
RECE_P	0.16 (0.128)	0.06 (0.595)	0.04 (0.677)	0.04 (0.666)	0.07 (0.491)	- 0.07 (0.482)	- 0.11 (0.266)	- 0.12 (0.241)	- 0.18 (0.084)	0.18 (0.072)
RLC_P	- 0.13 (0.210)	0.03 (0.808)	**- 0.30 (0.002)**	**- 0.36 (0.000)**	- 0.16 (0.120)	- 0.18 (0.077)	- 0.16 (0.116)	- 0.14 (0.171)	- 0.14 (0.180)	- 0.11 (0.272)
ROUND_P	**0.24 (0.001)**	**- 0.26 (0.010)**	0.18 (0.072)	0.17 (0.105)	**0.22 (0.035)**	0.16 (0.123)	**0.26 (0.011)**	**0.25 (0.015)**	- 0.04 (0.679)	- 0.10 (0.335)
SOR_P	0.01 (0.925)	- 0.07 (0.513)	**- 0.22 (0.031)**	- 0.19 (0.063)	**- 0.48 (0.000)**	- 0.01 (0.956)	0.06 (0.554)	0.06 (0.555)	- 0.05 (0.614)	- 0.12 (0.243)
STEREO_P	**0.22 (0.029)**	0.19 (0.064)	0.17 (0.090)	0.17 (0.106)	0.07 (0.474)	**0.21 (0.044)**	- 0.02 (0.813)	- 0.07 (0.529)	0.07 (0.485)	- 0.09 (0.380)
VAR_P	**0.32 (0.001)**	0.12 (0.244)	**0.22 (0.033)**	**0.27 (0.007)**	0.16 (0.129)	0.03 (0.747)	**0.28 (0.006)**	**0.29 (0.004)**	**0.29 (0.004)**	0.07 (0.525)
BFL_H	- 0.02 (0.881)	**0.46 (0.000)**	0.17 (0.091)	0.17 (0.091)	**0.25 (0.012)**	**0.29 (0.004)**	0.07 (0.485)	0.03 (0.752)	- 0.01 (0.893)	- 0.13 (0.211)
MAIL_H	- 0.01 (0.921)	0.08 (0.439)	- 0.03 (0.786)	- 0.03 (0.786)	- 0.09 (0.409)	0.13 (0.190)	0.16 (0.120)	0.15 (0.156)	0.04 (0.698)	0.12 (0.238)
ERS_H	0.06 (0.586)	0.02 (0.821)	**0.31 (0.002)**	**0.27 (0.007)**	**0.25 (0.014)**	**0.31 (0.002)**	0.12 (0.251)	0.08 (0.465)	0.08 (0.438)	**- 0.30 (0.003)**
FILT_H	0.00 (0.989)	0.04 (0.671)	**0.24 (0.016)**	0.16 (0.131)	0.16 (0.109)	**0.27 (0.008)**	- 0.03 (0.741)	- 0.08 (0.432)	- 0.01 (0.921)	- 0.09 (0.356)
DUR_H	0.19 (0.063)	0.01 (0.891)	0.16 (0.123)	**0.21 (0.041)**	0.14 (0.161)	**0.22 (0.031)**	**0.59 (0.000)**	**0.58 (0.000)**	**0.22 (0.029)**	- 0.03 (0.748)
RDUR_H	0.20 (0.055)	0.02 (0.861)	0.13 (0.190)	**0.21 (0.039)**	0.14 (0.163)	0.17 (0.102)	**0.60 (0.000)**	**0.61 (0.000)**	**0.23 (0.022)**	- 0.04 (0.713)
INR_H	- 0.08 (0.413)	- 0.16 (0.129)	- 0.16 (0.125)	- 0.10 (0.360)	0.04 (0.679)	**- 0.40 (0.000)**	- 0.15 (0.152)	- 0.10 (0.336)	- 0.04 (0.699)	**0.65 (0.000)**
MRS_H	- 0.11 (0.307)	**0.21 (0.038)**	- 0.12 (0.257)	- 0.06 (0.588)	- 0.02 (0.830)	0.04 (0.724)	- 0.02 (0.858)	- 0.03 (0.773)	- 0.01 (0.956)	- 0.05 (0.618)
ROUND_H	- 0.17 (0.094)	0.04 (0.737)	- 0.09 (0.390)	- 0.17 (0.107)	0.04 (0.700)	- 0.16 (0.108)	- 0.13 (0.190)	- 0.11 (0.269)	0.02 (0.852)	0.11 (0.302)
TEL_H	0.06 (0.569)	0.16 (0.116)	0.10 (0.353)	0.10 (0.353)	0.03 (0.777)	0.15 (0.155)	0.14 (0.174)	0.13 (0.217)	0.09 (0.387)	0.01 (0.963)
VAR_H	0.28 (0.054)	- 0.06 (0.560)	0.08 (0.448)	0.08 (0.448)	- 0.12 (0.231)	- 0.10 (0.331)	0.09 (0.360)	0.13 (0.202)	0.12 (0.232)	0.08 (0.439)

Table S2. (continued) Correlations between falsification indicators.

	MRS_h_P	MRS_m_P	MRS_l_P	ND_P	PRIM_P	RECE_P	RLC_P	ROUND_P	SOR_P	STEREO_P
MRS_h_P	1									
MRS_m_P	**0.72 (0.000)**	1								
MRS_l_P	**0.20 (0.050)**	0.18 (0.078)	1							
ND_P	**0.55 (0.000)**	**0.72 (0.000)**	0.19 (0.066)	1						
PRIM_P	- 0.02 (0.837)	0.00 (0.981)	- 0.03 (0.798)	- 0.07 (0.487)	1					
RECE_P	- 0.09 (0.395)	- 0.13 (0.218)	0.15 (0.137)	0.01 (0.930)	**0.30 (0.003)**	1				
RLC_P	- 0.11 (0.298)	- 0.20 (0.053)	0.02 (0.861)	**- 0.32 (0.002)**	0.16 (0.119)	0.15 (0.147)	1			
ROUND_P	- 0.00 (0.985)	- 0.00 (0.971)	0.11 (0.266)	- 0.05 (0.649)	0.10 (0.331)	0.16 (0.118)	- 0.11 (0.300)	1		
SOR_P	0.04 (0.696)	- 0.19 (0.065)	**- 0.30 (0.003)**	- 0.15 (0.153)	0.07 (0.472)	0.05 (0.610)	**0.28 (0.006)**	- 0.13 (0.196)	1	
STEREO_P	**0.25 (0.014)**	**0.23 (0.023)**	0.16 (0.128)	0.20 (0.053)	- 0.12 (0.243)	**0.22 (0.028)**	- 0.18 (0.085)	0.16 (0.114)	0.14 (0.185)	1
VAR_P	0.13 (0.224)	**0.23 (0.024)**	0.12 (0.226)	0.13 (0.193)	- 0.12 (0.231)	0.17 (0.101)	- 0.12 (0.226)	0.12 (0.255)	0.12 (0.228)	**0.36 (0.000)**
BFL_H	0.07 (0.473)	0.19 (0.067)	**0.37 (0.000)**	**0.24 (0.017)**	**- 0.24 (0.020)**	0.18 (0.074)	**- 0.22 (0.031)**	0.01 (0.959)	**- 0.36 (0.000)**	**0.40 (0.000)**
MAIL_H	- 0.02 (0.881)	0.02 (0.833)	- 0.02 (0.824)	0.14 (0.186)	0.04 (0.692)	0.08 (0.453)	- 0.08 (0.438)	0.07 (0.469)	0.09 (0.403)	0.01 (0.903)
ERS_H	**0.36 (0.000)**	**0.27 (0.007)**	0.15 (0.137)	**0.37 (0.000)**	0.00 (0.994)	0.10 (0.315)	**- 0.26 (0.011)**	0.14 (0.189)	0.02 (0.836)	**0.21 (0.042)**
FILT_H	0.19 (0.063)	0.16 (0.125)	0.03 (0.759)	0.15 (0.153)	- 0.06 (0.587)	- 0.17 (0.092)	**- 0.23 (0.026)**	0.08 (0.416)	**- 0.35 (0.000)**	0.02 (0.862)
DUR_H	- 0.02 (0.829)	0.07 (0.522)	0.07 (0.499)	0.18 (0.086)	0.01 (0.938)	- 0.03 (0.809)	- 0.11 (0.306)	**0.31 (0.002)**	- 0.16 (0.123)	- 0.08 (0.412)
RDUR_H	- 0.06 (0.580)	0.05 (0.646)	0.09 (0.394)	0.17 (0.092)	0.02 (0.872)	0.01 (0.951)	- 0.09 (0.396)	**0.30 (0.003)**	- 0.13 (0.209)	- 0.10 (0.348)
INR_H	- 0.16 (0.112)	- 0.10 (0.340)	0.17 (0.090)	**- 0.26 (0.010)**	0.12 (0.225)	0.06 (0.570)	0.06 (0.549)	- 0.11 (0.273)	**- 0.25 (0.015)**	- 0.15 (0.143)
MRS_H	0.01 (0.904)	- 0.10 (0.315)	0.11 (0.298)	0.10 (0.326)	- 0.13 (0.212)	**0.31 (0.002)**	0.10 (0.336)	- 0.12 (0.241)	**0.27 (0.007)**	0.15 (0.155)
ROUND_H	**- 0.21 (0.037)**	**- 0.21 (0.036)**	- 0.02 (0.833)	- 0.17 (0.106)	- 0.09 (0.369)	- 0.12 (0.236)	- 0.08 (0.445)	0.15 (0.148)	**- 0.29 (0.005)**	- 0.13 (0.198)
TEL_H	0.09 (0.369)	0.19 (0.063)	0.03 (0.797)	**0.21 (0.042)**	- 0.19 (0.068)	- 0.09 (0.411)	**- 0.22 (0.033)**	0.06 (0.561)	0.02 (0.840)	**0.27 (0.007)**
VAR_H	- 0.08 (0.448)	0.08 (0.417)	- 0.16 (0.129)	- 0.11 (0.307)	0.05 (0.603)	- 0.02 (0.837)	- 0.07 (0.480)	0.00 (0.974)	- 0.02 (0.879)	- 0.05 (0.646)

	VAR_P	BFL_H	MAIL_H	ERS_H	FILT_H	DUR_H	RDUR_H	INR_H	MRS_H	ROUND_H
VAR_P	1									
BFL_H	**0.36 (0.000)**	1								
MAIL_H	**0.24 (0.020)**	0.14 (0.186)	1							
ERS_H	0.05 (0.602)	0.12 (0.241)	- 0.08 (0.411)	1						
FILT_H	- 0.19 (0.068)	**0.37 (0.000)**	0.16 (0.130)	0.02 (0.882)	1					
DUR_H	**0.24 (0.017)**	0.17 (0.092)	**0.23 (0.027)**	0.00 (0.986)	**0.28 (0.005)**	1				
RDUR_H	**0.31 (0.002)**	0.14 (0.180)	0.18 (0.084)	0.01 (0.936)	0.10 (0.353)	**0.98 (0.000)**	1			
INR_H	- 0.16 (0.130)	- 0.06 (0.571)	- 0.01 (0.911)	**- 0.45 (0.000)**	- 0.03 (0.805)	- 0.03 (0.790)	- 0.03 (0.773)	1		
MRS_H	**0.24 (0.020)**	- 0.04 (0.722)	0.05 (0.637)	**0.37 (0.000)**	**- 0.53 (0.000)**	- 0.08 (0.426)	0.03 (0.780)	- 0.12 (0.245)	1	
ROUND_H	- 0.16 (0.113)	0.07 (0.508)	- 0.09 (0.390)	**- 0.21 (0.039)**	0.16 (0.113)	- 0.13 (0.212)	- 0.14 (0.161)	**0.26 (0.011)**	- 0.20 (0.245)	1
TEL_H	**0.25 (0.013)**	**0.23 (0.024)**	**0.53 (0.000)**	0.09 (0.409)	0.11 (0.272)	0.12 (0.248)	0.09 (0.369)	- 0.16 (0.109)	- 0.05 (0.656)	- 0.08 (0.419)
VAR_H	**0.26 (0.009)**	0.01 (0.960)	0.06 (0.580)	- 0.37 (0.000)	0.03 (0.763)	0.15 (0.135)	0.18 (0.081)	0.12 (0.237)	**- 0.26 (0.010)**	0.02 (0.850)

Table S2. (continued) Correlations between falsification indicators.

	TEL_H	VAR_H
TEL_H	1	
VAR_H	0.07 (0.482)	1

Notes: P-values (provided in parentheses) are based on a two-tailed significance test. Significant correlations ($p \leq 0.05$) are additionally marked in boldface.
Source: IAB-BAMF-SOEP Survey of Refugees in Germany (version SOEP.v33).
